# Drugs and Mental Health Problems among the Roma: Protective Factors Promoted by the Iglesia Evangélica Filadelfia

**DOI:** 10.3390/ijerph15020335

**Published:** 2018-02-14

**Authors:** Jelen Amador López, Ramón Flecha García, Teresa Sordé Martí

**Affiliations:** 1 CREA—Community of Research on Excellence for All, Facultat d’Economia i Empresa, University of Barcelona, Diagonal Nord, Diagonal 690, 08034 Barcelona, Spain; jelen.amador@ub.edu; 2 Department of Sociology, Facultat d’Economia i Empresa, University of Barcelona, Diagonal Nord, Diagonal 690, 08034 Barcelona, Spain; ramon.flecha@ub.edu; 3 Department of Sociology, Autonomous University of Barcelona, Edifici B, Office B3-179, 08193 Bellaterra, (Cerdanyola del Vallès), 08034 Barcelona, Spain

**Keywords:** Roma, Pentecostalism, drugs rehabilitation, mental health, social exclusion

## Abstract

*Background:* High incidences of drug consumption and mental health problems are found among the Roma population in Spain, a reality that remains understudied. Past studies have indicated the positive role played by the Iglesia Evangélica Filadelfia (IEF) in promoting rehabilitation and prevention of these practices. *Objective:* In this article, authors analyze in which ways the IEF favors processes of drug rehabilitation and mental health recovery as well as the prevention of these problems among its Roma members. *Methods:* A communicative qualitative approach was developed. It was communicative because new knowledge was created by dialogically contrasting the existing state of the art with study participants. It was qualitative because everyday life stories were collected, gathering the experiences, perceptions and interpretations of Roma people who are actively involved in three different IEF churches based in Barcelona. *Results:* This article identifies these protective factors: anti-drug discourse, a supportive environment, new social relations, role model status, the promotion of interactions, the revaluation of oneself, spiritual activities and the improvement of the feeling of belonging and the creation of meaning. *Conclusion:* The present research contributes new evidence to the current understanding of the role played by the IEF in improving Roma health status and how the identified protective factors can contribute to rehabilitation and recovery from such problems in other contexts.

## 1. Introduction

High levels of drug consumption and mental health problems are reported among the Roma population in Spain [1]. Research indicates that these two related problems are connected to social determinants of health, such as living conditions, employment, education and housing, among others [2,3]. Considering that EU official surveys show the Roma to be consistently found in unacceptable levels of deprivation, marginalization, and discrimination [4], these problems tend to persist within Roma communities, thus having not been well studied yet. While there is a relative wealth of data showing higher incidence of chronic diseases, infant mortality, less healthy lifestyles (which are related to nutrition problems, a greater number of accidents and the lack of access to health resources and preventive measures) [5,6,7], there is scarce research shedding light on strategies, factors or transformative dynamics signaling how to improve the Roma health status.

In this context, the increasing importance of the Iglesia Evangélica Filadelfia (IEF) and, more particularly its Pentecostal denomination, cannot be ignored. Based only in Spain, the IEF is part of the Christian movement known as Pentecostalism. The origin of Pentecostalism among the Roma dates back to the 1950s. In the north of France there is an evangelization movement initially headed by Clément Le Cossec (1921–2001) that led to the conversion to Pentecostalism of some Spanish Roma families living in that area. On their return to Spain, these families shared their conversion experience with other Roma families, and this is how Pentecostalism expanded among Spanish Roma. This evangelization movement led to the founding of the Iglesia Evangélica Filadelfia in Spain in 1969. In 2013, a total of 1100 congregations, between 150,000 and 200,000 members and between 3500 and 4000 pastors were reported throughout Spain [8]). For more than three decades now, this church has successfully penetrated the most remote Roma communities, incorporating many of the Roma cultural features into its organization and functioning, and gathering the largest number of Roma participants [9,10,11]. Previous researchers have indicated the role of the IEF in preventing certain habits and promoting healthy ones. These studies have focused on the effect of concrete prohibitions against consuming alcohol or drugs on the health of their members [12,13,14,15,16]. The Spanish Ministry of Health and Consumer Affairs has recognized the health benefits provided by the IEF, particularly its role in rehabilitating and preventing drug consumption [17]. Also, Estruch, Gómez, Griera and Iglesias [18] highlighted the work of the IEF in the prevention of addictions through the organization of campaigns to raise awareness and the promotion of detoxification in collaboration with Evangelical organizations that have rehabilitation centers [18]. 

The present article represents a step beyond previous studies, as it is aimed at disentangling whether there are factors that promote the rehabilitation and protect the Roma members of this church from drug consumption and mental health problems. In what follows, a literature review about Roma and drug consumption and mental health is presented. After, the methodological design is provided followed by the results section. The article ends with the discussion of the results and some concluding remarks. This research is framed under the work conducted by the authors under the IMPACT-EV project [19].

### 1.1. Greater Risk of Drug Consumption among the Roma

During the 80s, drug consumption and more particularly heroine hit the Spanish population, the Roma community being not an exception to it. This boom directly affected the core of many Roma families in Spain, not only as consumers but sometimes being taken advantage as traffickers by those who were making a business of it, leaving important damages behind. Due to this vulnerability, at that moment and at the present, research identifies a greater risk among the Roma of consuming drugs [1,2]. This greater exposure is identified in different countries. The Roma population is excluded and lives in marginalized neighborhoods, in unhealthy conditions where there are low levels of education, high unemployment rates and a high percentage of alcohol consumption and drug injection converge [1,2]. This is the case of Dzsumbuj, a neighborhood located in Budapest with a large Roma population. In 2004, a study was carried out in this neighborhood. While no cases of human immunodeficiency virus (HIV) were detected, the study drew attention to the high levels of HIV risk behavior in the study population, as indicated by high levels of drug injecting, the high prevalence of Hepatitis A Virus, Hepatitis B Virus and Hepatitis C Virus (HAV, HBV and HCV) infections, and the alarmingly high levels of hepatitis co-infections. The results identified that the risky behaviors among Roma or mostly Roma populations may put them at higher risk of contracting HIV and other blood-borne and sexually transmitted infections than the behaviors among mostly non-minority populations [2]. 

Similarly, in a study carried out in a juvenile detention center, it was identified in its sample that the highest percentage of acquired immune deficiency syndrome (AIDS) virus infections (70%) was concentrated among Roma drug addicts. It also warned of a high risk for said group to contract diseases such as hepatitis or tuberculosis. The consumption of alcohol, tobacco or injected drug increases this risk. If high poverty rates and unhealthy living conditions affecting the Roma population are combined, the risk for Roma people skyrockets. In fact, a tuberculosis outbreak was detected among the Roma in the Camp de la Bota (Barcelona), a marginalized neighborhood in Barcelona in 1985, which gave rise to a contact study. The tuberculosis incidence among the Roma people was 115 times higher than the tuberculosis incidence rate in Barcelona in the same year [1]. In addition, impoverished conditions and low levels of schooling, specifically among Romani women, are reported to have a negative impact on their ability to navigate the health system, leading to the proliferation of harmful practices and habits. In fact, studies indicate that Roma women with lower education tend to consume more drugs than those who have completed higher levels of education [7,20]. While relatively clear information is found about the incidence of drug consumption among Roma communities, very little knowledge has been generated on the specific processes of rehabilitation. The present article is meant to expand on this gap. 

### 1.2. Mental Health Problem Incidence among the Roma

Research has already shown that experiences of discrimination and social exclusion are detrimental to mental health. While a reactive system responds to social exclusion by activating defense mechanisms, social support stimulates reward mechanisms. Defense mechanisms activated in response to episodes of exclusion induce intense stress responses with detrimental effects on mental health. However, supportive networks can reverse defensive reactions, such as startling, or inhibiting the negative effects of depression [3]. Despite these clear-cut connections, little research has been dedicated to exploring mental health problems among the Roma. Along similar lines, although recent studies [3,21] have proven the negative impact of the consequences of the financial crisis, and more particularly, house evictions on the mental health among the Spanish people, the impact of exclusion and poverty on the mental health of the Roma community has not yet been contemplated. Studies looking at mental health problems among the general population show the highest risk of depression or anxiety among women [3], and an even a higher incidence is reported among Roma women compared to non-Roma women [7]. For unhealthy habits, Roma women stand out for the highest percentage of alcohol consumption and the lowest rate of physical activity. However, there is evidence that they smoke less for cultural reasons [7]. If there is little information about the incidence of mental health problems within Roma communities, no research effort before has focused on identifying which particular processes can contribute to combat it. It is precisely this gap that the present research aims at fulfilling.

## 2. Materials and Methods

### 2.1. Communicative Methodology Design

A communicative qualitative study design was selected to conduct the present research. The communicative methodology [22] allows for the establishment of an egalitarian dialogue between the researchers and the study participants, leading to a process of dialogic creation of knowledge. While the former are responsible for providing scientific knowledge, the investigated people contribute their ways of life [23], their experiences, their life trajectories and their ways of understanding the world. This communicative paradigm opens the doors of research to groups such as the Roma people, who have often been excluded from the processes of the creation of scientific knowledge [24], and it has been identified by the European Commission as being especially suitable for working with disenfranchised communities [22,25].

The authors followed a qualitative strategy to collect data, as it was necessary to capture the perceptions, experiences and opinions of those members of the church who have been exposed to cases of detoxification and mental health problems. The fieldwork has been developed during 2017.

### 2.2. Participants

Fieldwork was conducted in three churches belonging to the IEF during 2017. Two of these churches are located in Barcelona neighborhoods; they have been operating for more than 20 years and at the moment they count approximately with 100 members. The last one is a more recently created church (4 years ago), located in the outskirts of Barcelona, and it has approximately 30 members. The choice of Barcelona is due to the fact that it stands out as one of the pioneer cities in hosting the Pentecostal movement among the Roma people in Spain [26]. These three sites served as the spaces from which we recruited all our participants. 

Our participants were all selected purposively. We were seeking for study participants who meet the following four criteria: (i) Being of Roma descent; (ii) to be a member of one of the churches, seeking for people who have been recently involved and those who have been there for years, and with different responsibilities within the Churches. Pastors were given priority because their experience and position have given them access to many examples of overcoming both drug addiction and mental health problems. The pastors’ wives (*pastora*) were also interviewed because they play a key role in meeting the needs of the women in the church, act as role models in the congregation and organize activities for women; (iii) had experienced themselves or knew someone close to them who had been drug-addicted or had had mental health problems; and (iv) seeking maximum diversity in terms of gender, age, level of education, family situation. In total, nine communicative everyday life stories were performed, all recorded and debriefed. Confidentiality was guaranteed throughout the research regarding how data is kept, based on the Data Protection Directive, meaning that research has been conducted by respecting the integrity of the stated objectives, allowing all the participants to have access to their own data, and to be able to withdraw from the study at any time they wish. All these conditions are contained in the consent forms as well as a full explanation of the overall goals of the present project. Related to the storing, the team saves all the data regarding individuals in electronic format in the investigators’ locked office. Finally, all the researchers are committed to avoiding any illicit use of the released data, conclusions or political recommendations. They will intervene, and ultimately take appropriate action, to correct any kind of misinterpretation or misuse of their work [27]. Pseudonyms have been used all throughout. In the Table 1 below is a brief description of the profile of each of the participants identified by pseudonyms:

### 2.3. Data Collection

Communicative everyday life stories (CELS) were used. The CELS consists of a collaborative dialogue between the people investigated and the researchers to reflect together on specific situations relating to the concept being studied [28].

### 2.4. Analysis

According to the Communicative Methodology, the analysis of the information is based on identifying the transformative and exclusionary dimensions of the target reality. While the former are all those elements that tend to promote rehabilitation and to recover from mental health problems, the latter are all those elements that contribute to maintain to mental health problems and drug abuse. The present article presents all those elements identified as the protective factors that allow detoxification and positive mental health.

## 3. Results

In Table 2, a summary of all the identified protective factors are presented. Although for the purpose of clarity, we have separated those more connected to drug consumption from those more related to mental health, our data indicates that they are all strongly connected.

### 3.1. Detoxification

The resources that the IEF provides for combating addictions stood out especially during the 1970s and 1980s when the consumption of heroin was booming, so the impact on detoxification was very noticeable. Although there has been a decrease in the consumption of heroin and other drugs in the Roma community, rehabilitation processes are still in need.

#### 3.1.1. Anti-Drug Discourses: From Contamination of the Body to Illegal Activity

In our analysis, a set of anti-drug discourses have been identified in the three churches that are recognized as playing a key role by our study participants. The first one consists of perceiving one’s own body as a temple of the Holy Spirit, so it becomes essential not to contaminate the body with harmful substances such as drugs. The consumption of drugs is, therefore, forbidden, since it represents an attack on the integrity of the body. This is one of the most recurrent arguments already highlighted in the literature that explains why drugs are prohibited [12] within this denomination. The incorporation of these norms in the day-to-day of the members of the IEF contributes to overcoming addictive behaviors, and thus, to initiating detoxification processes. The same rationale also prevents church members from committing suicide, as will be argued in the next section.

Our subjects’ testimonies shared how their conversion process implied the internalization of a second important anti-drug discourse, that is, how drug trafficking is considered to be incompatible with being part of the IEF, because it is an illegal activity. For this reason, among many other respondents, Pedro not only gave up his addictions but also stopped drug trafficking. Pedro and Carmen trafficked drugs, which in their case meant that both had been sentenced to prison. This rehabilitation goes beyond health and promotes a radical transformation in their new lives.

Thus, a third anti-drug discourse is widespread, which is how social recognition is connected to respect due to adherence to Pentecostal norms. Therefore, someone who does not respect the norms will not have a positive image within the IEF and the Roma community in general. This is the case of those who engage in drug consumption or trafficking, and therefore, the effect of the social control is to persuade people to stop such practices. In these cases, penalization measures are envisaged, such as not receiving communion, the sacrament in which those publicly baptized participate during Sunday services. Likewise, they will not be able to hold positions of responsibility such as that of pastor, since they are contaminating “their temple.”

Jonathan is a 60-year-old Roma man, married, father of 4 children and a grandfather. He has been participating in the IEF for 37 years, 32 years of which have been as a pastor. He is part of the group of Roma men who experienced the conversion process during the 1980s (at the start of the IEF) when he was a drug addict, and after detoxifying, he became a pastor.

“God made you free, he has given you the freedom to choose, he does not force you, but he calls you. The Iglesia Evangélica Filadelfia is a delegation of God on earth to call you, and through the Iglesia Evangélica Filadelfia God calls you to put your life in order and to get out of the existing mud that brings the sin that there is into the world and that poverty often makes you embrace, because poverty sometimes makes you embrace sin in order to survive. The Church that announces and proclaims a message of cleansing is the voice of God, so that believing it, you can come out from the bad and unhealthy habits.”

(Jonathan, 60 years old)

It should be noted that a similar process is also recognized beyond the church, reaching the Roma community in general. This connects with the idiosyncrasy of the ethnic minority, which, regardless of economic status or level of education, gives greater recognition to people who respect Roma values. Our study participants explained how since their conversion they have accomplished a personal revaluation incorporating the Pentecostal norms, among which were the rejection of drug consumption, the condemnation of violence and the abandonment of illegal practices.

As part of these anti-drug discourses, and recognizing groups at risk such as the youth, the three Churches organize a wide range of leisure activities such as football matches, prayers for the children, Bible studies or dinners aiming to keep the youth engaged and active there, moving away from harmful contexts and risky circles. The anti-drug discourse is embedded in many activities, including those oriented toward the children. Samara is a 35-year-old Roma woman, who is a female pastor, and emphasizes the importance of educating children about rejecting drugs. In her narrative, she refers to the case of her 11-year-old son:

“My son is being raised in the Gospel which is a healthy environment, in which drinking or getting high with drugs is not often seen, nor going out at night and returning at the end of the morning, because in my house it would be inconceivable that my son would do that—never say never—but likewise my child and the children around, too, know that God demands that they stay clean, because our body is a temple of the Holy Spirit, so we have to keep it clean. It is something that God demands of us, and our children grow in this environment.”

(Samara, 35 years old)

#### 3.1.2. Supporting Environment

For many of our study participants, their initial involvement in the church represented entering a supportive environment that allowed them to make crucial decisions in their lives. One example of this was Jonathan, who clearly identifies his participation in the IEF as a key element in overcoming his drug addiction. Jonathan identifies in his conversion process a personal transformation that has allowed him to change the course of his life. Most of his previous friends died due to drug addictions or are in prison for illegal activities. He is convinced that had he not joined the IEF, his fate would have been that of his friends. Jonathan described his first contact with the IEF and how he found the needed conviction to giving up drug consumption:

“I realized that the road I was taking was not the right one, and when I arrived at the Iglesia Evangélica Filadelfia, I met people who had gone through my own personal situation and some of them had come out of the anguishing situation in which I found myself, and they were for me a sort of a mirror where I looked at myself. I also found support from people who felt sorry for me and who God put in my way to help me out, to know how to advise me, to have patience (...) and with the help of God and with the love and affection of the brothers and sisters I was able to get out of that dark cave I was in.”

(Jonathan, 60 years old)

Jonathan’s words highlights the support network that is represented by the community of church members. Both cooperation and complicity are key factors for the consolidation and success of the rehabilitation processes. The affection and support found in the community help to reverse the isolation, loneliness and social refusal they have previously faced. In this regard, Jonathan notes that the support of the brothers and sisters in the church helped him out of “the dark cave” where he was.

Isaac is a 25-year-old young Roma man who has been a pastor in the IEF for a year. He says that even though he has never been a drug addict, he has had contact with drugs since very early ages. In fact, he admitted to having smoked cannabis when he was 12, and when he was 13, he snorted cocaine 3 or 4 times. Therefore, there was a high degree of risk that Isaac would end up becoming a drug addict as many of his peers did. One of his brothers died because of drugs. However, he notes that his active participation in the church since he was 15 acted as the impetus that allowed him to get away from drugs:

“At 15 years old, I was not a rascal but I was a sort of a dissolute lad, and I was aware that this would not end well (...) A Roma pastor entered the church and simply bet on me. To be baptized in the Iglesia Evangélica Filadelfia, you are asked for some requirements that show you have converted to the Gospel, that you see a change in your life. The pastor came and baptized a few young people from the church, except me. At that time, I smoked, I wore earrings, I was a scoundrel... He (the pastor) had been working with me for quite awhile and he had very strong feelings for me, which always I observed, and talked to me about God and that I would regret it and that I could not continue like this. This man spent 15 months as a pastor in the church, and at least every time he saw me he said, ‘Man, you have to convert, you have to start following God, your house is a Christian house and you will end up very badly.’ I did not pay attention to him, but his words remained in my subconscious, and I thought he was right. In addition, I remember that once he explained to me an anecdote that he imagined that I was walking along the edge of a wall, and he told me you are walking, but at any moment you fall and hurt yourself, stop walking around here and start walking on the mainland. All those words remained with me in my subconscious.”

(Isaac, 25 years old)

For Isaac, the pastor’s trust and support were crucial for him to make the decision to be baptized and to change the course of his life path, giving drugs and tobacco up in order to actively participate in the church. Isaac notes that for him it was very important that his pastor believed in him and that he could change. It is important to consider the impact of this support for this young Roma, who comes from depressed contexts with high levels of drug consumption among his peers. The church represented for this youth an alternative social space where new relations, new habits and role models could be found.

#### 3.1.3. Becoming Role Models

The church is full of people’s stories of personal transformation, and thus, these persons become role models for the newcomers. They serve as examples for the rest of the members of the church and promote a life pattern away from drugs. Thus, those who are in the process of transformation are highly motivated to become role models and to prove to the community their effort to move away from difficult situations.

Next, we find Isaac’s father Pablo’s story. Pablo was a man with a serious cocaine addiction. He also began to participate in the IEF during the 1980s and represents another example of the many detoxifications that occurred at that time. Isaac notes that his father was an orphan at a very early age, and it hurt him a lot because the father figure is very important in the Roma community. When he lost his behavior model, Pablo began to take cocaine very often. With a serious addiction, he joined the IEF and began his detoxification process, which he successfully completed in six months. Once detoxified, Pablo began his training process to become a pastor, and with that, his story became an example to follow within the church. Isaac speaks about his father’s rehabilitation process:

“My father at 31 was a young rascal and a crazy lad with no one around to stop him; he started ingesting cocaine every day and he got addicted, he was hooked for many years, and this had repercussions for gender violence on top of many other problems at home (...) I have not lived it but they explained it to me that my father was nervous (...) My father went to my mother’s town because he wanted to have different friends because he believed that would change him. When my grandmother arrived here, she was a very good Christian and always spoke to him about God. They preached the Gospel to her at church. It was not until my father decided to give God a chance, what in fact occurred was that God was giving it to him. In addition, it completely changed my father. In only 6 months, my father was already raised from candidate to pastor.”

(Isaac, 25 years old)

#### 3.1.4. New Social Relations

The last protective factor identified is connected to the improvement of relationships within the family as well as beyond. This is the case with Pedro, a 47-year-old Roma man who has been participating in the IEF for approximately 8 years. Pedro started very young in the world of drugs and had been taking drugs for 20 years when he first started participating in the church. His partner, Carmen, describes the impact on Pedro’s health:

“My life changed completely. When I observed him, he was also sick, and I observed that with the worship his illness was gradually disappearing. Until there arrived a moment when the doctor said that Pedro was cured, and that it appeared to be a miracle. He had Crohn’s disease and because of that, he gave up medication. From then onwards, there has been a tremendous change at home. I cannot believe it, the way he was and how is he now.”

(Carmen, 49 years old)

Parallel to his detoxification, Pedro explains his recovery from Crohn’s disease as he was back to medication. Thus, due to his new life, he also recognizes improvements of his emotional relationship with Carmen. Both refer to more prudent behavior, a rejection of violence, a better use of economic resources and an improvement in trust in the couple:

“A complete change, I had been hooked on drugs for 20 years, whereas today I do not even smoke. Then, in my life, I thought that I would never see myself like this, because I said, I will be 80 and still be like this, however I do not even smoke, so I have given up all my addictions. I do not leave my house, I go out in the morning for a while to the park, for a while with the birds, then to my worship and from here to my house and I do not go out anymore (...) For me, the biggest thing is that I was able to give up all drugs, tobacco, methadone, I have left everything. I think that today, I still do not believe it, you think that you have been hooked for 20 years, and here since I have been in the cult for 6 or 7 years, it has cleaned me up completely. Not a pill to sleep, not even diazepam, that normally that everybody takes it currently, not even that I take to sleep.”

(Pedro, 47 years old)

Improvements in his relationships have been reported beyond his marriage. Pedro explains how he made new friendships, and thus, he stopped seeing those with whom he used to consume drugs and going to these places. Pedro notes that he moved from being surrounded by drug addicts to getting associated with people who participated in the church and who have a clear rejection of drugs. This new socialization process is also a factor of social pressure that reinforces the change of lifestyle and, consequently, the processes of detoxification. However, the fact of integrating into the congregation, assuming and internalizing Pentecostal values and creating meaning that represented their participation in the church have been sufficient to achieve detoxification.

### 3.2. Mental Health

#### 3.2.1. Promoting a Wellbeing/Happiness Discourse

Many of the values and habits spread by the three analyzed churches are aligned with healthy mental wellbeing. Our participants highlighted how their participation represented a reinforcement of their self-esteem. Thus, Pentecostal values emphasize the importance of kindness or bravery, downplaying insecurities. Religious beliefs directly collide with considering committing suicide at any point, and support and companionship is displayed to ensure that people do not consider it as an escape route from their situation. Rebecca, a 43-year-old Roma woman, shared the case of her sister-in-law, a woman with depression, who at the time she received the news that her youngest daughter had leukemia, decided to commit suicide. However, she explains that the constant support in the church was crucial for her:

“Well, they care a lot for her, ‘Calm down’ they say, and they give her advice, ‘that the Lord is with you, you have to get out of this, look at the miracle that God has done to your daughter’ and they are with her, and they advise her. How do you think that this whole process would have been with your daughter’s illness if she had not been in church? She said it with these words, ‘I would have gone to the 13th floor and jump out.’”

(Rebecca, 43 years old)

Some of the core values of the church act as a protective factor against suicides. For instance, there is the belief that there is life after death, and therefore, members of the church who live according to Christian ethics aspire to be welcomed into the Kingdom of Heaven after they die. However, for those who commit suicide, the expectation is different, as they would be denied entry into the Kingdom of Heaven. This conviction plays a crucial role in suicide attempts among church members, especially in the face of the loss of a loved one. When Rebecca ‘sister in law suffered the death of her daughter, the conviction that those who commit suicide cannot reunite with their loved ones in the Kingdom of Heaven led her to desist from her suicidal plans.

When a case at risk for depression in the church is identified, this support network sets in motion a number of measures. Samara narrates her mother’s case. She was at the time immersed in a deep depression that led her to consider taking her life. Her participation in the church and in spiritual activities has led to clear improvements in her mental health. She has even abandoned the medication that kept her in bed to regain her normal rhythm of life.

During the religious service and in the interactions between the members of the church, there is an emphasis on the improvement and empowerment of oneself. They cope with the fears and complexes by counting on a “God Almighty” and the members of the church who intervene as a guarantee of success. This speech has a significant impact on the expectations and the day-to-day lives of the members of the IEF. As already mentioned, pastors, because of the spiritual authority they hold, are recognized as crucial points of support in difficult times. Jonathan explains that his brother-in-law came to him because he felt that this would be his last night. It is usual for prayers to be held together as a show of support and to give more strength to the petition. Jonathan describes how, through the prayers that he offered with his brother-in-law, he managed to overcome the feeling of panic that had invaded him. Providing support and companionship when someone is in trouble are resources identified to favor wellbeing and overcoming depression.

Samara described the situation of a young girl with agoraphobia. Since she is the pastor’s partner, Samara plays a crucial role in the care of women. She gets involved in solving problems that go beyond participation in the church. Likewise, the activity of the pastors contributes to healthiness within the congregation.

“A girl had a phobia about going out, and I noticed that when I talked to her, it wasn’t me, I noticed that I was speaking on behalf of the Holy Spirit, and every time I spoke to her, the girl felt better. She called me on the phone and explained what was happening to her at that moment, so I started talking to her about God, and she was right on the street and said ‘Pastor, thank goodness you have spoken to me, how much goodness you have given me’ and she carried on with her shopping, with her life.”

(Samara, 35 years old)

This companionship and advice can contribute to overcoming panic situations associated with mental problems such as anxiety or depression.

#### 3.2.2. A Sense of Belonging

Religious services occur every day. Bearing in mind that most Roma women are unemployed, their daily attendance at church is the activity that breaks with the routine of domestic chores and family responsibilities. Thus, our respondents recognize that it forces them to take care of their image, to relate to other people beyond the family, and thus, that it has a direct positive impact on their self-esteem and personal well-being. Samara describes how the support network they find in the church helps them to better face personal and family problems and to cope with stressful situations connected to poverty or social exclusion with hope and more positive attitudes. Samara describes what advantages for the mental health of Roma women she finds in participating every day in the church:

“Making up yourself every afternoon, leaving your environment, the monotony, I do not know … the change of our daily environment, going out every evening to congregate with people, to talk to one another.”

(Samara, 35 years old)

The positive interactions add up and reinforce the self-esteem of the participants. Samara described how participating in the church has helped her appreciate some of her virtues. At the same time, it has allowed her to develop skills such as public speaking.

“I’ve been a very self-conscious girl. People told me I was beautiful, and I looked ugly, but really, I felt it, I was ashamed to speak in a loud voice, I did not have the security or the freedom to speak in front of a group of people and … I kept quiet, I did not speak and in the church you live with many people, spiritually God is truly showing you what you are worth, what we are worth, that it does not matter what your looks are but what you have in your heart, yearnings that you have for God, how brave you can be in the hands of God, because all that helped me overcome my complexes, and today, I can say that I am a person free to speak.”

(Samara, 35 years old)

## 4. Discussion

Evidence has been found of how the participation of Roma in the three studied IEF churches has contributed to the rehabilitation throughout the success of numerous detoxification processes and to the prevention of drug consumption. The IEF spread a set of protective factors that have been identified as key elements for the detoxification process, namely anti-drug discourse, a supportive environment, new social relations, and role models.

Our data shows how these three churches become supporting environments to initiate radical personal transformations, as well as to generate new healthier relationships, and make our respondents feel like role models who are proud of their personal achievements and who are motivated to help others to become so. All these elements make these churches the most effective spaces for detoxification that have existed throughout these years for the Roma in Spain. This is also consistent with previous research that had already noted this role [10,17,18]. In this paper, we move beyond the previous research to present a systematic analysis of particular dynamics and discourses that have become protective factors.

The social activities organized by the churches have been shown to be beneficial in preventing young people from being in contexts where they are at risk of drug use. Similarly, Adamczyk and Felson [29] illustrated how the participation of adolescents in activities organized by religious institutions, such as sports, have a positive impact on their health. They found feelings of wellbeing, a better physical condition, a reduction in the consumption of alcohol and drugs, as well as a delay in the age of initiation to sex. Our results are thus consistent with these studies, contributing to the wealth of research that shows how religious participation tends to encourage healthy habits [29] and therefore prevent risky behaviors.

The churches analyzed here, besides being social spaces where preventive interactions occur, all contribute to spreading an anti-drug discourse that keeps their members away from addictions and drug use [9,10]. Thus, the IEF tends to project an ideal of a Roma individual who lives according to Roma culture and Christian values and who refuses drugs and any illegal activity. This ideal type has penetrated beyond the church and has also been embraced by other members of the Roma community. This success has triggered the proliferation of detoxification since the beginning of the penetration of the IEF into the Roma community in Spain in the early 1980s. This role has been maintained and strengthened throughout the years.

These churches are also safe spaces in which mental health problems are approached, a finding that is also aligned with previous research [30]. Nevertheless, the specific benefits of participation in the IEF for the mental health of the Roma minority have not been explored yet. The present paper fills this gap, and we inquired about the benefits of participation in the IEF regarding the counteraction of the negative effects of exclusion on the mental health of the Roma people. Social engagement is vital for the survival of many species. When people are socially excluded or have a greater sensitivity to rejection, four fundamental needs are shown to be affected: belonging, self-esteem, control and meaningful existence, which are required for human survival and effective social functioning [31]. Here, we argue that the IEF enhances their members’ feeling of belonging, in this case, in the Pentecostal community. According to research, the feeling of belonging improves the conception of one’s own social identity [32,33]. This is a particularly interesting aspect in minority groups such as the Roma community, who experience a strong burden of stereotypes, discrimination and rejection. All these advantages reverse the negative effects of exclusion such as isolation or loneliness that can lead to depression or anxiety [32,33]. Furthermore, the importance of reinforcing self-esteem and self-confidence has been outlined as crucial for our respondents’ lives. They all agree that since their participation in the church, they felt more kind and brave, and thus, they all recognize improvements in their communication-related skills. It has been noted how the development of communication skills improves interactions and mental health while encouraging empowerment and freedom.

In the same vein, a longitudinal analysis in Taiwan recognized that participation in religious movements or secular activities organized by religious institutions promoted an improvement in mental health and reduced symptoms of depression [32]. Recent empirical works on suicide [34,35] show that greater social support and attachments are related to fewer depression symptoms and other mental health issues among very diverse populations. Improvement in the feeling of belonging and promotion of interactions and social cohesion among those who profess religious beliefs suggests a lower percentage of depression, suicide attempts, and feelings of anguish and a higher level of wellbeing [36,37,38,39]. Thus, research indicates that people active on a religious level present a better state of health than those who are inactive [33]. Our research adds consistency to this argument by showing that active participation in religious movements is related to the promotion of a healthy lifestyle, with the consolidation of support networks and the development of strategies to cope with stress in the best way possible. All these advantages are clearly related to the improvement of mental health. In addition, this participation increases social cohesion within the community, overcoming loneliness and contributing to wellbeing. Particularly, we noticed preventive factors for depression and suicide in the analyzed IEF. The daily participation in the church implies leaving home and breaking with the routine of many Roma women who mostly (not all) devote themselves only to domestic chores. It is well recognized that an improvement in taking care of one’s image benefits self-esteem and wellbeing. Additionally, participation in the church enhances interactions and promotes networks of solidarity and support. Besides all these factors, our research is also consistent with recent studies that highlight the crucial role of churches in reinforcing positive attitudes towards standard medical treatments [40]. In the particular case analyzed here, our testimonies highlight how their participation in the church helped them to follow medical assistance; however, they also recognize how a minority of members would not fully agree with these statements. This is an unexplored reality that should be further pursued in future studies.

## 5. Conclusions

There is still a long way to go to fully flesh out the crucial role played by the IEF within the Roma community in Spain. This article is an attempt to shed light on its particular role in rehabilitation and preventing drug consumption and mental health-related problems. We note the role of the three analyzed churches as transformative platforms that have resulted in an improvement of their members’ lives. The successful acceptance of the Pentecostal denomination by the ethnic minority also justifies that the contributions of the IEF have transcended beyond their members to be adopted also by the Roma community in general. Therefore, the healthy lifestyle promoted by the Pentecostal denomination has also had an impact on the prevention of drug use more broadly in the Roma community. Similar effects have occurred with other excluded minorities when adhering to Pentecostalism [15]. These three churches have also represented a springboard for the social participation of the Roma community. This is particularly important if we bear in mind that the ethnic minority faces numerous barriers to their participation in social life [41,42,43], a process that might positively correlate with being away from drugs and being mentally healthy.

The identified factors against drug addictions and mental health problems have been identified. Banning of the use of drugs is the main norm to explain the effect of these three churches belonging to the IEF on the processes of detoxification of the ethnic minority. Beyond this norm, support networks, socialization against drugs, and positive role models are identified as protective factors. Thus, the promotion of a healthy lifestyle that overcomes exclusion also affects mental health problems. Therefore, preventative factors affecting depression, suicide attempts, poor self-esteem, and phobias have also been recognized. These factors contribute to the promotion of interactions, the consolidation of support networks, the reevaluation of oneself and the development of skills and competencies that foster the improvement of personal self-confidence. The IEF discourse breaks with the stereotype that links the Roma identity with exclusion, poverty and criminality. In contrast, it presents an ideal model of a Roma person who follows the values of Pentecostal ethics but also Roma values.

The present research represents only the tip of an important iceberg still understudied. There are still many related aspects both regarding processes that improves health status of the Roma, as well as the role played by the IEF within it, which have never been systematically approached. Their future analysis would contribute to reinforce those transformative and protective factors that make possible for an increasing number of Roma individuals and families to carry on the flourishing lives they wish to have.

## Figures and Tables

**Table 1:** Study participants’ profile.

Pseudonym	Position	Profile Description
Rebecca	Female Pastor	A 43-year-old married Roma woman who is a mother of 5 children and a grandmother. Rebecca works as a street vendor and lives with her husband and three of her children. She was baptized when she was 12 years old and has been a female pastor for approximately 12 years. Rebecca has closely experienced the lives of her sister and sister-in-law, two women with depression problems who participate in the IEF.
Jonathan	Pastor	Jonathan is a 60-year-old Roma man who is married, a father of 4 children, and a grandfather. He is an old-age pensioner who lives with his wife and one of his daughters. Jonathan has been participating in the IEF for 37 years and served as a pastor for 32 years. He experienced a process of detoxification himself.
Pedro	None	Pedro, a 47-year-old Roma man is married to Carmen, 49. Together, they have two children, and they are grandparents. Both are ex-convicts, and they have been participating in the IEF for about eight years. When they started, Carmen had already left prison and Pedro was still on parole. After 20 years of consuming drugs, Pedro decided to abandon drugs completely.
Carmen	None	
Isaac	Pastor	Isaac is a 25-year-old young Roma man who has been a pastor in the IEF for a year. He is married with three children ages 6, 4 and 2 years old. He has attended church since he was a child because he was raised in a Christian family, and his father is a pastor, too. Isaac is a street vendor, and has now entered college. Isaac says that he is the youngest of four brothers and that, although he has never been a drug addict, he has had contact with drugs since a very early age.
Samara	Female Pastor	Samara is a 35-year-old Roma woman. She is married with three children ages 16, 11 and 5 years old. She lives with her husband and her three children. She is a street vendor and would like to be a psychologist. She left school at the age of 11 and is currently participating in a university access course for people over 25. She has closely experienced the case of her mother, a woman with depression problems who has sporadically participated in the church.
Rocío	None	Rocío is a 23-year-old Roma woman who lives with her partner, is pregnant and has a daughter from a previous relationship. She participated in the IEF as a girl with her family. Currently she is out of work, and she has completed basic studies. She has overcome depression.
Isabel	Female Pastor	Isabel is a 44-year-old Roma woman; she is married, a mother of five children and a grandmother. She lives with her husband and three younger children. She has been a female pastor for approximately twelve years. She works in the cleaning sector. She has been participating in the IEF for 20 years.
Ana	None	Ana is a 45-year-old married Roma woman and the mother of three children, ages 11, 10 and 6. She has always been dedicated to street vending, but right now she does not work. She has finished basic studies. She began to participate in the IEF at the age of fifteen. She says that she started because she has always had nervous problems, and her participation in the Pentecostal denomination helped her mental health, including during serious problems such as having her daughter diagnosed with leukemia.

**Table 2:** Summary of the Protective Factors.

Protective Factors	
Detoxification	Anti-drug discourse
	Supportive Environment
	Becoming role models
	New social relations
Mental Health	Promoting wellbeing/happiness discourse
	A sense of belonging
